# Proposed treatment algorithms for dogs with chronic bronchitis associated with irreversible airway changes: bronchiectasis and/or bronchomalacia

**DOI:** 10.3389/fvets.2025.1686007

**Published:** 2025-11-14

**Authors:** Aurélie Lyssens, Elodie Roels, Cécile Clercx, Frédéric Billen

**Affiliations:** Department of Clinical Sciences, Faculty of Veterinary Medicine, University of Liège, Liège, Belgium

**Keywords:** bronchi, cough, therapy, respiratory, canine

## Abstract

Chronic bronchitis (CB) in dogs involves persistent inflammation of the bronchial walls and excessive mucus production within the airways, with or without bronchial infection, and may lead to degenerative airway changes such as bronchiectasis (BE) and bronchomalacia (BM). Standardized treatment protocols for CB with concurrent BE and/or BM (BEBM) are lacking. This article proposes a therapeutic approach for dogs with CB and BEBM, based on veterinary literature and relevant human medical data. Two treatment algorithms are outlined, depending on the presence or absence of cytological evidence of bacterial infection in bronchoalveolar lavage fluid (BALF) and/or bronchial brush samples. For cases with suspected infection, indicated by intracellular bacteria on cytology, first-line therapy with oral doxycycline is recommended pending BALF culture and quantitative polymerase chain reaction (qPCR) results. If warranted, antibiotic therapy should be escalated stepwise after culture/qPCR confirmation, in accordance with antimicrobial stewardship principles. In non-infectious inflammatory cases, inhaled glucocorticoids are advised as first-line therapy and may also be used in infectious cases unresponsive to antibiotics alone. Mucoactive agents and cough suppressants are not recommended in the initial protocol but may be considered as adjunctive, symptom-targeted treatments on a case-by-case basis, avoiding unnecessary or unsupported interventions. These proposed algorithms are not intended as definitive clinical guidelines, but as a starting point for discussion and future validation. They emphasize rational and prudent use of antibiotics, alone or alongside anti-inflammatory therapy, to improve patient outcomes while minimizing antimicrobial resistance risks. Further research is needed to assess the long-term efficacy of this approach.

## Introduction

Chronic bronchitis (CB) in dogs is characterized by persistent inflammation of the bronchial walls and excessive mucus production within the lumen of the airways, with or without concurrent bronchial infection, and may progress to degenerative airway changes such as bronchiectasis (BE) and bronchomalacia (BM) (1–4). BE is characterized by a progressive and irreversible dilatation of the bronchial wall while BM is defined as a regional to diffuse dynamic airway collapse of segmental bronchi, subsegmental bronchi, or both (1–4). BE is reported in both human and canine patients, while BM is mostly observed in canine medicine and only sporadically described in human paediatric cases (4, 5). The pathogenesis of both BE and BM remains unclear, but current evidence suggest they result from a complex interplay of chronic infection, inflammation, degenerative changes and impaired mucociliary clearance (2, 6, 7).

Chronic bacterial infection is a hallmark of BE in humans, significantly influencing disease progression (8). Currently, no standardized clinical guidelines define the therapeutic approach for managing BE and BM in humans or dogs (4, 5, 9). Treatment guidelines for human BE, such as those from the European Respiratory Society (ERS, 2017) and the British Thoracic Society (BTS, 2019), rely primarily on expert opinion and low-quality evidence from small randomized trials (10, 11). Current medical management of BE and BM in humans and dogs includes antibiotics, oral or inhaled anti-inflammatory agents, antitussives, bronchodilators, antimuscarinic agents, or mucolytics, though robust evidence supporting their efficacy is lacking (4, 5, 10). Despite limited evidence of bacterial infection in canine BE and/or BM, antibiotics are often used empirically, raising antimicrobial stewardship and One Health concerns (1, 4, 12, 13).

Based on the current available knowledge from veterinary and human literature, the aim of this article is to propose two standardized treatment protocols for dogs affected with CB and airways changes including BE and/or BM with or without concurrent airway bacterial infection.

## Reaching a diagnosis

The diagnostic process for CB associated with BE and/or BM in dogs requires a systematic approach to confirm these conditions while excluding other common causes of chronic cough that may necessitate specific treatments, such as cardiac disease, neoplasia, parasitic infection, or tracheal collapse (3). Moreover, known underlying causes of BE, such as aspiration-related lung injury, inhaled foreign bodies, parasitic or fungal infections, idiopathic eosinophilic bronchopneumopathy, idiopathic pulmonary fibrosis, and primary ciliary dyskinesia should be considered and systematically ruled out. Such diseases require a specific diagnostic and therapeutic approach, which is beyond the scope of this article. For an in-depth description of the diagnostic process, readers are directed to other relevant publications (1, 4, 7, 13–20). The diagnosis of BE and BM is ideally established using a combination of diagnostic imaging, such as computed tomography, together with bronchoscopy (1, 4). Based on bronchoscopy, dogs diagnosed with BM can be subclassified into three subcategories based on a previously described grading system: 25%–50% reduction in airway diameter, 50%–75% reduction in airway diameter and >75% reduction in airway diameter (4). Results of quantitative polymerase chain reaction (qPCR) assays on bronchoalveolar lavage fluid (BALF) for lower respiratory pathogens including *Bordetella bronchiseptica* (Bb), *Mycoplasma cynos* (Mc), *Angiostrongylus vasorum*, and *Crenosoma vulpis* may aid in diagnosis. It is important to note that the causal relationships between CB, BE, and/or BM in dogs remain unclear. While CB-related inflammation may contribute to BE and BM, the reverse is also plausible. Many canine BE cases remain idiopathic (1). Therefore, the associations described in this study should be interpreted with caution. Dogs without cytological signs of inflammation [total cell count (TCC) < 600 cells/μL and neutrophil percentage in BALF <12% of TCC] are outside the scope of this protocol. Also, patients with BE or BM and concurrent bacterial pneumonia were not included, as these conditions represent a distinct disease process requiring a different diagnostic and therapeutic approach.

## Proposal of treatment approaches

**Figure 1:**
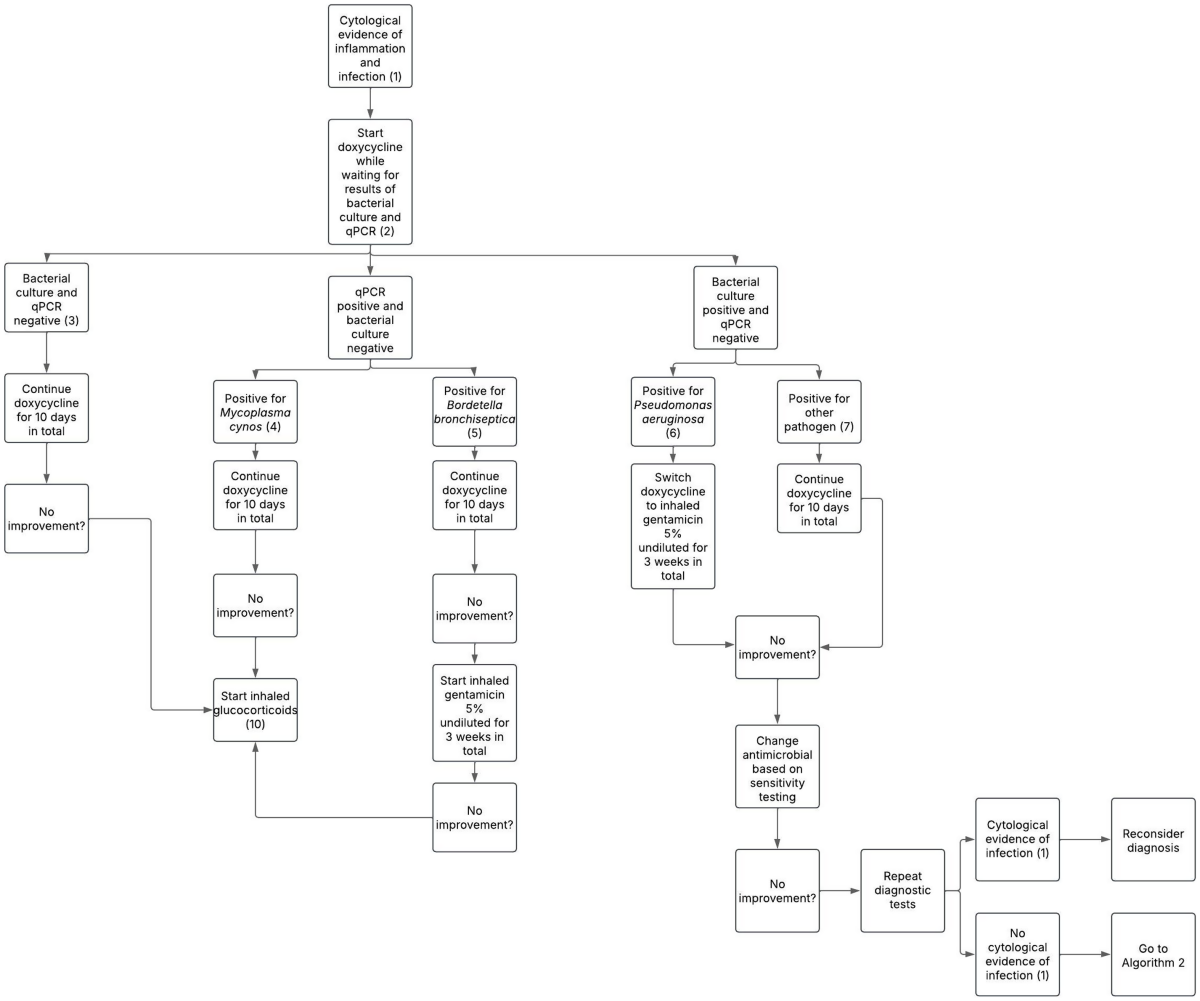
Proposed treatment protocol for dogs with chronic bronchitis associated with bronchiectasis and/or bronchomalacia in the presence of overt infection based on bronchoalveolar lavage fluid cytology. Numbers in parentheses refer to explanatory notes within the main text.

**Figure 2:**
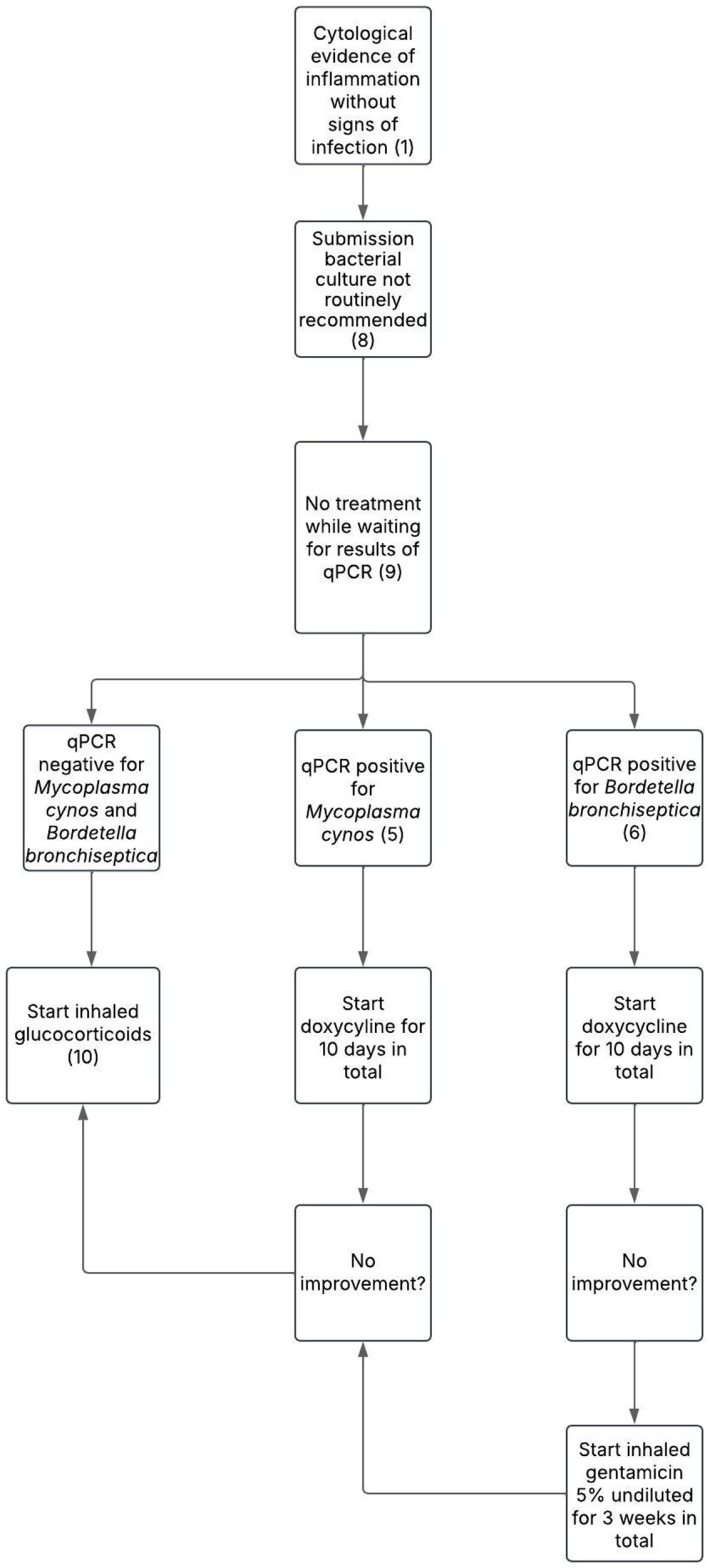
Proposed treatment protocol for dogs with chronic bronchitis associated with bronchiectasis and/or bronchomalacia in the absence of overt infection based on bronchoalveolar lavage fluid cytology. Numbers in parentheses refer to explanatory notes within the main text.

The present proposal was developed through consensus among 4 diplomates of the European College of Veterinary Internal Medicine (ECVIM), each with experience in respiratory clinical practice (ranging from 5 years to over 30 years). The proposal includes two distinct treatment algorithms: one for dogs with CB and evidence of BE and/or BM where bacterial infection is confirmed based on BALF and/or bronchial brushing cytology (Figure 1), and another for non-infectious inflammatory cases (Figure 2). This distinction reflects the necessity of tailoring clinical management. The numbered steps in brackets in the algorithm correspond directly to the clinical criteria and treatment considerations detailed in the main text, and should be interpreted in conjunction with it. For clarity, throughout this section, the term ‘improvement’ is used to describe a reduction in the frequency and severity of clinical signs, together with an improvement of the dog’s quality of life.

Cytological evidence of bacterial infection, evaluated either in-house when expertise is available on site or via an external laboratory, is primarily based on the identification of bacteria within the cytoplasm of airway neutrophils (intracellular bacteria) on cytological evaluation of BALF and/or bronchial brushing. When possible, bacteria should also be classified either using Gram-coloration or bacteria morphology, as this can provide useful preliminary information about the likely bacterial pathogens involved and interpretation of bacterial culture results. This criterion, used to distinguish infectious from non-infectious cases, forms the basis for selecting between the two proposed treatment algorithms (Note 1). It is essential to interpret cytological findings in the context of any recent or ongoing antimicrobial treatment, as prior antibiotic use may suppress visible cytological indicators of infection and lead to false-negative interpretations. Clinical judgment and further diagnostics should guide management in these cases.

If overt signs of infection are observed on BALF and/or bronchial brush cytology, initiating antimicrobial therapy promptly is recommended to prevent complications or disease progression. Doxycycline (10 mg/kg SID), the first-line empirical treatment for bacterial bronchitis recommended by the International Society for Companion Animal Infectious Diseases (ISCAID) guidelines, is effective against a wide range of canine respiratory pathogens and is associated with a low incidence of adverse effects (21) (Note 2).

In cases of negative culture and qPCR results on BALF, a 10-day course of doxycycline is recommended (21). False-negative results can occur due to prior antibiotic use, which may suppress or eliminate bacterial growth, reducing microbial load below detection thresholds (22). Sampling limitations such as insufficient BALF volume or collection from unaffected lung regions may also compromise diagnostic sensitivity (23). Laboratory errors such as processing delays or suboptimal transport conditions are additional sources of false-negative results (23). A positive clinical response within 7–10 days supports a diagnosis of bacterial infection; antibiotic treatment should then be continued for 1 week beyond clinical resolution (21). Conversely, lack of improvement suggests that infection is unlikely the primary cause of the clinical signs (Note 3).

A positive qPCR result for Mc despite negative culture results, is not unexpected as Mc requires specialized culture media but can be reliably detected by qPCR (24). A Ct value below 34 is considered a positive result (25). Doxycycline is recommended as the first-line antimicrobial treatment for Mc infection in dogs (Note 4) (21). If no improvement occurs, treatment may be extended or the diagnosis reconsidered, including repeating diagnostic tests to confirm the presence of Mc or identify other respiratory pathogens (21). Fluoroquinolones and azithromycin have shown *in vitro* efficacy against *Mycoplasma* spp. and resistance to doxycycline has been documented in some *Mycoplasma* species in other animals (26–28). However, no resistance to doxycycline in canine *Mycoplasma* species has been reported (29). It is important to note that some *Mycoplasma* spp. may be found in the lower airways of healthy dogs, suggesting they can be part of the commensal microbiota (30). However, Mc is rarely identified in healthy individuals and is more consistently associated with respiratory disease, indicating its likely pathogenic role (30). Lack of clinical response to doxycycline suggests that inflammation, rather than infection, is the predominant cause of symptoms. This is frequently observed in chronic airway diseases such as BE and/or BM, where non-infectious inflammation may persist. In these cases, treatment should be redirected toward inhaled glucocorticoids (Note 10). Other *Mycoplasma* species, such as *Mycoplasma canis*, were not considered in this algorithm due to its currently unproven pathogenic role in canine respiratory disease (31).

Doxycycline is also recommended as the first-line antimicrobial treatment for Bb infection in dogs, following the same dosage regimen as for Mc (21) (Note 5). However, studies suggest that doxycycline may not achieve therapeutic concentrations at the apical surface of the bronchial epithelium, limiting its effectiveness in treating infections within the airway lumen (32). Aerosolized delivery of 3-5 mL (depending on the speed of the nebulizer to aerosolize the product) of 5% undiluted gentamicin via a mask during a minimum of 10 min, twice daily for 3 weeks, has been shown to effectively induce clinical cure in dogs with Bb infection (33). For small dogs, nebulization can alternatively be performed by placing the animal in a closed container or box covered with a towel to retain the aerosol. Additionally, a 3-min inhalation protocol was investigated, confirming its efficacy and safety (34). If there is no improvement following treatment with oral doxycycline and subsequent inhaled gentamicin, inflammation rather than active infection is likely responsible for the clinical signs and treatment with inhaled glucocorticoids is recommended (Note 10).

*Haemophilus influenzae* and *Pseudomonas aeruginosa (P. aeruginosa)* are among the most common pathogens identified in humans with BE, with *P. aeruginosa* being linked to more severe disease and frequent exacerbations (8). Given its clinical impact and intrinsic resistance profile, targeted management is required when this organism is detected (35). In cases of *P. aeruginosa* infection, defined as clinically relevant growth exceeding 1.7 × 10^3^ colony-forming units per milliliter (CFU/mL) of BALF (36), doxycycline should be stopped and switched to inhaled aminoglycosides since *P. aeruginosa* is intrinsically resistant to tetracyclines (37) (Note 6). In humans with BE, current treatment guidelines for *P. aeruginosa* infections recommend inhaled aminoglycoside antibiotics or, if not tolerated, macrolides (10, 11). Treatment duration typically ranges from 6 weeks to 3 months (10, 11). In this proposal, a 3-week treatment is recommended, followed by reassessment. If improvement is absent or partial, the antimicrobial regimen should be adjusted based on culture and sensitivity testing, prioritizing oral drugs with proven efficacy in penetrating the blood-bronchus barrier (BBB), such as macrolides, fluoroquinolones, tetracyclines and lincosamides (21, 38). If no improvement is observed, diagnostic tests, such as repeat imaging and BALF analysis, should be repeated to confirm *P. aeruginosa* persistence, detect other pathogens or reveal other pathologies. Treatment can be extended up to 6 weeks, with evaluations and repeat testing every 3 weeks as clinically indicated.

For bacterial infections not caused by *P. aeruginosa*, based on a positive culture exceeding the clinical relevance threshold of 1.7 × 10^3^ CFU/μL (36), first-line treatment in humans consists of oral macrolides such as azithromycin or erythromycin, followed by inhaled gentamicin or doxycycline (10, 11). Based on this rationale, continuation of doxycycline is recommended while monitoring the patient’s clinical response (Note 7). If no improvement is seen after 7–10 days, the antimicrobial regimen should be tailored based on culture and sensitivity testing, prioritizing oral drugs known to effectively penetrate the BBB (21).

If no intracellular bacteria are seen on BALF cytology and/or bronchial brushing (Note 1), bacterial culture is generally not recommended, particularly when cytological evaluation can be performed immediately on-site by experienced personnel, as this allows rapid triage and avoids unnecessary costs. Conversely, in the absence of on-site cytological expertise, it is advisable to submit samples for both cytology and bacterial culture immediately. This ensures that, if intracellular bacteria are later identified on external cytology review, the corresponding sample has already been processed for culture, thereby avoiding delays and potential loss of bacterial viability (Note 8). In either case, antimicrobial therapy should be withheld pending qPCR and/or bacterial culture results to support antimicrobial stewardship principles (39) (Note 9). Importantly, a positive bacterial culture alone, without cytological evidence of infection, does not confirm infection, as the lungs are not sterile and bacteria may be present without causing disease (40).

If bacterial infection is excluded and qPCRs are negative, inhaled glucocorticoids should be initiated (Note 10). In human medicine, anti-inflammatory use in BE is controversial (10, 11). ERS and BTS guidelines caution against long-term corticosteroids due to unclear benefits and frequent side effects (10, 11). Similarly, there is no evidence supporting their use in either human or canine BM (4, 5). However, glucocorticoids have been shown to be effective in managing CB in dogs (3, 4, 41). Given that CB frequently coexists with BE and BM, particularly in the canine population, the use of corticosteroids may still be appropriate to target the inflammatory component. While oral glucocorticoids provide rapid short-term relief, symptoms often relapse after discontinuation, necessitating long-term use (41). Prolonged oral therapy carries a risk of systemic side effects (42). Although inhaled formulations may also lead to systemic side effects with long-term use, they are generally preferred in CB (41). Fluticasone propionate by inhalation via a spacer, with a starting dose of 1 puff of 100 μg twice daily for 6 to 8 breaths in dogs weighing less than 20 kg, and 2 puffs of 100 μg (total dose 200 μg) twice daily for 6 to 8 breaths in dogs weighing over 20 kg should be considered (41). In the long-term, the dose may be gradually tapered every 3–4 weeks based on the dog’s clinical response to determine the optimal dosing regimen to control the clinical signs without causing substantial side effects (41). Although most studies have focused on this dry aerosol formulation delivered via a spacer, other inhaled corticosteroids (e.g., budesonide) may also be used, depending on availability and clinical preference (43, 44). However, literature on this subject in veterinary medicine is lacking.

## Discussion

This study proposes two structured treatment algorithms for dogs with CB associated with BE and/or BM, providing a practical approach based on the cytological presence or absence of bacterial infection. These protocols support evidence-based clinical decision-making, emphasizing the judicious use of antimicrobials and glucocorticoids, aiming to optimize treatment outcomes minimizing unnecessary medication use.

Determining true infection and the need for antibiotic therapy in dogs with BE and/or BM remains a clinical challenge, particularly due to overlap in BALF characteristics cytological findings between infectious and non-infectious inflammation. In this study, infection is defined as the concurrent presence of intracellular bacteria on BALF cytology and/or bronchial brushing, thereafter supported by a positive bacterial culture and/or qPCR results. This multimodal definition aims to increase diagnostic confidence while minimizing unnecessary antimicrobial use. However, each of these markers alone has limitations. For instance, cytology may underestimate infections: intracellular bacteria were found in only 32%–79% of culture-confirmed BALF samples in dogs with lower respiratory disease (15, 36, 43). Furthermore, pathogens such as Mc and Bb are poorly visualized on cytology due to their structural characteristics (15, 33). In these cases, bronchial brushing or qPCR may enhance detection (25). Similarly, relying solely on semiquantitative bacterial culture thresholds can be misleading. A recent study showed that although a cut-off of 1.7 × 10^3^ CFU/mL is commonly used to define clinically significant bacterial growth, nearly half of the dogs with clinical and cytological signs of infection had lower CFU counts (45). Moreover, dogs with high CFU counts but lacking cytological signs of infection may be colonized rather than infected (40). Therefore, this approach uses the 1.7 × 10^3^ CFU/mL threshold as a reference point, but not as a strict criterion. Importantly, cultures resulting solely from enrichment procedures should be interpreted with caution, as they may reflect contamination rather than true infection (46).

The use of inhaled glucocorticoids in non-infectious cases is a key component of the proposed treatment protocol. Although there is currently no evidence-based data supporting their efficacy in CB cases associated with BE and/or BM, their benefit has been demonstrated in cases of CB without dynamic airway collapse (41). Given that CB commonly coexists with BE and/or BM, and shares similar inflammatory mechanisms, it is reasonable to expect a therapeutic benefit in these patients as well. This expectation is further supported by the clinical experience of the authors, who have observed improved outcomes and quality of life in affected dogs treated with inhaled glucocorticoids.

Other medications, such as mucoactive agents, cough suppressants, and bronchodilators, are not included in the proposed treatment protocols due to limited and inconclusive evidence supporting their routine use in dogs with CB and BE and/or BM (4). However, cough suppressants may be considered for non-inflammatory forms of CB (4). While mucoactive agents may theoretically help with thick mucus and cough suppressants may alleviate severe cough, both of which can significantly affect quality of life, their efficacy remains unproven, and guidelines from human medicine do not strongly support their use (4, 5, 10, 11). Therefore, these treatments are reserved as adjunct options outside the standard protocols presented here.

Despite its structured approach, this protocol has limitations. A key limitation of this work is that the proposed protocol arises from joint discussions among a small group of 4 ECVIM diplomates, all with a particular interest in respiratory medicine. While it reflects expert clinical opinion, it does not yet represent a broader consensus. Establishment of a multi-institutional panel in order to critically evaluate and validate the relevance and applicability of this protocol would be interesting. Secondly, the proposed protocol relies mainly on limited veterinary literature and extrapolations from human medicine. It is primarily based on the authors’ clinical expertise, reflecting expert opinion rather than strong, high-level evidence. Another limitation is that access to equipment and expertise for BALF analysis is generally restricted to university or specialized centers, limiting the applicability of the proposed algorithm to well-equipped referral practices.

To conclude, this protocol emphasizes judicious antibiotic use, targeted bacterial infection management, and anti-inflammatory treatment, while minimizing antibiotic resistance risks. It is ready for use in future prospective studies in dogs with CB associated with BE and or BM to assess its clinical safety and efficacy. These studies should not only evaluate clinical outcomes but also consider quality of life. The protocol may furthermore be applicable to cases of CB without degenerative airway changes.

## Data Availability

The original contributions presented in the study are included in the article/supplementary material, further inquiries can be directed to the corresponding author.
